# Molecular profiling identifies synchronous endometrial and ovarian cancers as metastatic endometrial cancer with favorable clinical outcome

**DOI:** 10.1002/ijc.32907

**Published:** 2020-02-18

**Authors:** Casper Reijnen, Heidi V.N. Küsters‐Vandevelde, Marjolijn J.L. Ligtenberg, Johan Bulten, Marloes Oosterwegel, Marc P.L.M. Snijders, Sanne Sweegers, Joanne A. de Hullu, Maria C. Vos, Anneke A.M. van der Wurff, Anne M. van Altena, Astrid Eijkelenboom, Johanna M.A. Pijnenborg

**Affiliations:** ^1^ Department of Obstetrics and Gynaecology Radboud University Medical Center Nijmegen The Netherlands; ^2^ Department of Obstetrics and Gynaecology Canisius‐Wilhelmina Hospital Nijmegen The Netherlands; ^3^ Department of Pathology Canisius‐Wilhelmina Hospital Nijmegen The Netherlands; ^4^ Department of Pathology Radboud University Medical Center Nijmegen The Netherlands; ^5^ Department of Human Genetics Radboud University Medical Center Nijmegen The Netherlands; ^6^ Department of Obstetrics and Gynaecology Elisabeth‐TweeSteden Hospital Tilburg The Netherlands; ^7^ Department of Pathology Elisabeth‐TweeSteden Hospital Tilburg The Netherlands

**Keywords:** endometrial neoplasms, ovarian neoplasms, synchronous tumors, molecular pathology, clonality

## Abstract

Synchronous primary endometrial and ovarian cancers (SEOs) represent 10% of all endometrial and ovarian cancers and are assumed to develop as independent entities. We investigated the clonal relationship between endometrial and ovarian carcinomas in a large cohort classified as SEOs or metastatic disease (MD). The molecular profiles were compared to The Cancer Genome Atlas (TCGA) data to explore primary origin. Subsequently, the molecular profiles were correlated with clinical outcome. To this extent, a retrospective multicenter study was performed comparing patients with SEOs (*n* = 50), endometrial cancer with synchronous ovarian metastasis (*n* = 19) and ovarian cancer with synchronous endometrial metastasis (*n* = 20). Targeted next‐generation sequencing was used, and a clonality index was calculated. Subsequently, cases were classified as *POLE* mutated, mismatch repair deficient (MMR‐D), *TP53*‐wild‐type or *TP53*‐mutated. In 92% of SEOs (46/50), the endometrial and concurrent ovarian carcinoma shared at least one somatic mutation, with a clonality index above 0.95, supporting a clonal origin. The SEO molecular profiles showed striking similarities with the TCGA endometrial carcinoma set. SEOs behaved distinctly different from metastatic disease, with a superior outcome compared to endometrial MD cases (*p* < 0.001) and ovarian MD cases (*p* < 0.001). Classification according to the TCGA identified four groups with different clinical outcomes. *TP53* mutations and extra‐utero‐ovarian disease were independent predictors for poor clinical outcome. Concluding, SEOs were clonally related in an overwhelming majority of cases and showed a favorable prognosis. Their molecular profile implied a primary endometrial origin. *TP53* mutation and extra‐utero‐ovarian disease were independent predictors for outcome, and may impact adjuvant systemic treatment planning.

## Abstract 2

**What's new?**

When primary endometrial and ovarian tumors are found simultaneously in the same patient, it has been assumed that they are separate cancers that developed independently. However, in this study, the authors found that these tumors share a clonal origin 92% of the time. They also found that these “synchronous” cancers tend to have a favorable prognosis, with far better outcomes than metastatic disease. Some subgroups, including *TP53* mutations and extra‐utero‐ovarian disease, were independent predictors for poor clinical outcome, which may impact adjuvant treatment planning.

**Abbreviations**
CI: clonality index
DSS: disease‐specific survival
EEC: endometrioid endometrial cancer
FFPE: formalin‐fixed, paraffin‐embedded
MD: metastatic disease
MMR‐D: mismatch repair‐deficient
MSI: microsatellite instability
OC: ovarian cancer
ProMisE: Proactive Molecular Risk Classifier for Endometrial Cancer
SEO: Synchronous primary endometrial and ovarian cancer
smMIP: single‐molecule Molecular Inversion Probe
TCGA: The Cancer Genome Atlas
TSG: tumor suppressor gene
VAF: variant allele frequency

## Introduction

The co‐occurrence of carcinomas in the endometrium and ovary can point toward either the presence of independent synchronous primary endometrial and ovarian cancers (SEOs) or metastatic disease (MD), with the endometrium or ovary being the primary origin.^1^, ^2^, ^3^ The Scully criteria are used to distinguish SEOs from MD based on histopathological features, for example, histologic similarity, size, the presence of precursor lesions, location and invasion pattern (Supporting Information Table S1).^4^ SEOs typically behave as independent primary tumors and are characterized by good prognosis.^5^, ^6^, ^7^ SEOs can have both endometrioid and nonendometrioid histology.^8^ Patients diagnosed with stage I endometrioid endometrial cancer (EEC) and synchronous Stage I endometrioid ovarian cancer (OC) have a comparable survival to patients diagnosed with Stage I EEC alone.^9^ The distinction between two independent early‐stage SEOs and MD is important, as it directly impacts adjuvant treatment planning. Even though these cancers have histopathological features that help discriminating them from MD, recent series have shown that most SEOs are actually clonally related.^10^, ^11^, ^12^, ^13^, ^14^ Whether the endometrium or the ovary could be designated as the primary origin remains to be elucidated.

The Cancer Genome Atlas (TCGA) provided a molecular prognostic classification of endometrial cancer (EC) based on mutational profile, identifying an “ultramutated” subgroup associated with mutations in the exonuclease domain of *POLE* and an excellent prognosis; a “hypermutated” subgroup with microsatellite instability (MSI) and an intermediate prognosis, a “copy‐number high” subgroup with *TP53* mutations and an unfavorable outcome, and a copy number‐low subgroup with no specific molecular profile and an intermediate prognosis.^15^

In our study, we investigated the clonal relationship between endometrial and ovarian carcinomas in a large cohort of SEOs and metastatic carcinomas classified according to histological criteria. The molecular profiles were compared to TCGA data to explore primary origin. Subsequently, molecular profile was correlated with outcome, as this might impact adjuvant therapy.

## Materials and Methods

### Patients

This multicenter study consisted of two previously published well‐documented retrospective study cohorts investigating the prognosis of patients with SEOs from the Radboud university medical center Nijmegen and the Elisabeth–TweeSteden Hospital Tilburg.^5^, ^6^ All patients were surgically treated between 1996 and 2009. For this current study, additional patients treated between 2010 and January 2018 were identified. All patients had concurrent endometrial and ovarian tumors at the time of diagnosis. All carcinomas were histopathologically reviewed by a pathologist with special interest in gynecology (A.v.d.W. or J.B.) using the Scully criteria.^4^ Based on the revision according to the Scully criteria, three categories of patients were distinguished: patients treated for SEOs; patients treated for EC with synchronous ovarian metastasis (endometrial MD); and patients treated for OC and synchronous endometrial metastasis (ovarian MD). Patients' characteristics, clinical presentation, surgical treatment, adjuvant therapy and follow‐up data were obtained from the medical records. The study was approved by the Medical Ethics Committee of the Radboud university medical center (number 2018‐4342) and performed according to the Code for Proper Secondary Use of Human Tissue (Dutch Federation of Biomedical Scientific Societies, http://www.federa.org).

### Immunohistochemical analysis

Immunohistochemical analysis of the mismatch repair (MMR) proteins PMS2 and MSH6 was performed.^16^ In short, blank 4 μm formalin‐fixed, paraffin‐embedded (FFPE) sections were cut on Superfrost+ glass slides. After antigen retrieval with EnVision FLEX High pH Target Retrieval Solution, and blocking of endogenous peroxidase with hydrogen peroxide, all slides were incubated with anti‐MSH6 (clone EPR3945 1:400, Abcam, Cambridge, UK) or anti‐PMS2 (clone A16‐4 dilution 1:20, BD Biosciences, San Jose, CA). Subsequently, they were incubated with EnVision FLEX and visualized with High pH visualization system according to the manufacturer's instructions for use. Counterstaining was performed with hematoxylin, and the slides were dehydrated and mounted. Mismatch repair deficiency (MMR‐D) was defined as total loss of nuclear staining of a MMR protein, in the presence of a positive internal control.

### DNA extraction

Representative areas of EC and OC tissue in the surgical specimens were marked and selected by means of microdissection from 2 × 20 μm thick FFPE sections. The tumor cell percentage was estimated from the marked tumor areas. These specimens were digested overnight at 56°C in TET‐lysis buffer (10 mmol/l Tris/HCL pH 8.5, 1 mmol/l EDTA pH 8.0, 0.01% Tween‐20) with 5% Chelex‐100 (Bio‐Rad, Hercules, CA) and 0.2% proteinase K, with subsequent inactivation at 95°C for 10 min. After centrifugation, the supernatant transferred into a clean tube. DNA concentration was determined using the Qubit Broad Range Kit (Thermo Fisher Scientific, Waltham, MA).

### smMIP design and library preparation

The samples were analyzed with single‐molecule Molecular Inversion Probes (smMIPs). The design of the smMIPs (Integrated DNA Technologies, Leuven, Belgium) as well as the library preparation were previously published.^17^ The panel consisted of (regions of) eight genes important for EC and OC oncogenesis (*ARID1A*, *CTNNB1*, *KRAS*, *MTOR*, *PIK3CA*, *PTEN*, *POLE* and *TP53*). All smMIPs were designed in a tiling manner for hotspots in oncogenes and all coding as well as splice site consensus sequences of tumor suppressor genes (TSGs), with preferential targeting of both strands by two independent smMIPs (Supporting Information Table S2). The smMIP probes are constructed by an extension and ligation probe arm (40 bp long) with a 112 bp gap and a common backbone sequence for PCR‐based library amplification. The ligation probe arm and backbone are connected by means of an 8 bp degenerate sequence (8xN) serving as a Unique Molecular Identifier (UMI, “single‐molecule tag”). Next, the smMIP probes were mixed and phosphorylated with 1 μl of T4 polynucleotide kinase (M0201; New England Biolabs, Ipswich, MA) per 25 μl of 100 μmol/l smMIPs and ATP‐containing G4 DNA ligase buffer (B0202, New England Biolabs). The molecular ratio between gDNA and smMIPs was set at 1:3,200 for each individual smMIP and the standard genomic DNA input was set at 100 ng. Next, a capture mix was made (volume 25 μl) with the phosphorylated smMIP pool, 1 unit of Ampligase DNA ligase (A0110K; EpiBio, Madison, WI) and Ampligase Buffer (A1905B, DNA ligase buffer), 3.2 units of Hemo Klentaq (M0332; New England Biolabs), 8 mmol of dNTPs (28‐4065‐20/‐12/‐22/‐32; GE Healthcare, Little Chalfont, UK) and 100 ng of genomic DNA in a 20 μl volume. This capture mix was then denatured at 95°C for 10 min and subsequently incubated for probe hybridization, extension and ligation at 60°C for 18 hr. After cooling, to perform exonuclease treatment, Exonuclease I (10 units; M0293; New England Biolabs) and III (50 units; M0206; New England Biolabs) and Ampligase Buffer was added to the capture mix (total of 27 μl) and the mix was incubated at 37°C for 45 min, with subsequent inactivation at 95°C for 2 min. Twenty microliters was used for PCR in a total volume of 50 μl including a common forward primer, bar‐coded reverse primers, and iProof high fidelity master mix (1725310, Bio‐Rad, Veenendaal, the Netherlands). The resulting PCR products were then pooled and purified with 0.8× volume of Agencourt Ampure XP Beads (A63881, Beckman Coulter, Woerden, the Netherlands).

### Sequencing and analysis

The purified libraries, denatured and diluted to 1.2 pmol/l, were then sequenced on a NexSeq500 device (Illumina, San Diego, CA) using the manufacturer's instructions (300 cycles High Output sequencing kit, v2), resulting in 2x150bp paired‐end reads. All resulting Bcl files were converted to fastq files and bar‐coded reads were then demultiplexed. Single‐molecule‐directed assembly of the duplicate reads was performed generating consensus (‘unique’) reads with the software Sequence Pilot (version 4.4.0; JSI medical systems, Ettenheim, Germany). Variant detection thresholds for variant calling in Sequence Pilot were set at 1% of all unique reads at that specific position and a minimum of five unique reads representing ≥3 individual gDNA molecules. Variants were annotated as “drivers”, “potential drivers”, “mutations of unknown significance”, “likely benign” and “benign” as described in Richards *et al*., using amongst others publicly available databases such as The Clinical Knowledgebase (CKB, https://www.jax.org/clinical-genomics/ckb), ClinVar (https://www.ncbi.nlm.nih.gov/clinvar/), Cancer Genome Interpreter (CGI, https://www.cancergenomeinterpreter.org/home), the Catalog of Somatic Mutations in Cancer (COSMIC, cancer.sanger.ac.uk/cosmic).^18^ Only the former three categories were taken into consideration and included known activating hotspot mutations for the oncogenes, and frameshift, nonsense, missense and splice‐site mutations for the included tumor suppressor genes. Synonymous mutations were only considered when present at exon ends. Intronic mutations were excluded with the exception of splice site sequences. To determine whether sufficient DNA molecules were sequenced to reliably exclude mutations above a certain mutant allele frequency with a certainty of >95%, a cumulative binomial distribution was used that calculated the required unique read depths.^17^ These required read depths were assessed in the context of the estimated tumor load (percentage of neoplastic cells dissected estimated with microscopy). The molecular subgroups were assigned according the previously published Proactive Molecular Risk Classifier for Endometrial Cancer (ProMisE) criteria, distinguishing four groups: *POLE* mutated, MMR‐D, *TP53*‐wild‐type and *TP53*‐mutated.^19^, ^20^, ^21^ In contrast to the published algorithm, assignment to the *TP53‐*mutated group was based on sequencing results instead of p53‐immunohistochemistry, correlating in an excellent way.

### Statistical analysis

Clinicopathological differences between subgroups were compared using the Fisher's exact test and χ^2^ for discrete variables and the Mann–Whitney U test for continuous variables. To estimate whether the SEOs were actually clonally related, an earlier published clonality index was used to quantify the likelihood of two carcinomas sharing mutations not expected to have co‐occurred by coincidence.^10^, ^11^ This clonality index adjusts for the frequency of a given mutation, as hotspot mutations can be highly recurrent. To correct for this, frequencies retrieved from the TCGA data portal were used (https://portal.gdc.cancer.gov/).

The clonality index (CI) was defined as

$$\mathrm{CI}=\left\{\begin{array}{c} 1-\prod_{k=1}^{n}f_{k},n>0\\ 0,n=0 \end{array}\right.$$

In this formula, *f*_k_ is the percentage of endometrioid endometrial carcinomas from TCGA harboring a given mutation and *n* is the number of shared mutations between a pair of synchronously diagnosed carcinomas. Clonality indices were calculated twice, since the primary origin is unknown: based on frequencies retrieved from the 2013 TCGA endometrial carcinoma tumor set (*n* = 240), and based on frequencies retrieved from the 2011 TCGA ovarian carcinoma tumor set (*n* = 316; retrieved from www.cbioportal.org).^15^, ^22^, ^23^

The frequencies of mutated genes were compared between the three subgroups using χ^2^. To investigate whether the primary origin of the SEO subgroups could be inferred based on molecular similarities, the molecular profiles from the SEO subgroup were compared to the molecular profiles from the TCGA 2013 endometrial carcinoma tumor set using χ_2_.^15^, ^22^

Kaplan–Meier curves were constructed for disease‐specific survival (DSS) comparing patients with a diagnosis of SEO, endometrial MD and ovarian MD. Also, Kaplan–Meier curves were constructed based on molecular subgroup. DSS was calculated from the date of primary treatment to the date of death caused by the disease or, for surviving patients, to the date of the last follow‐up. The log‐rank test was used. Univariable and multivariable Cox regression analysis explored associations between potential predictors and DSS, including histology, age (<70; ≥70 years), adjuvant therapy, the presence of extra‐utero‐ovarian disease, and ProMisE subgroup. Extra‐utero‐ovarian disease was defined as disease other than the endometrial and ovarian carcinoma (e.g., pelvic/para‐aortic lymph nodes, omentum, peritoneum).

### Data availability

Data used for this analysis are available upon reasonable request to the corresponding author.

## Results

### Patients

**Figure 1:**
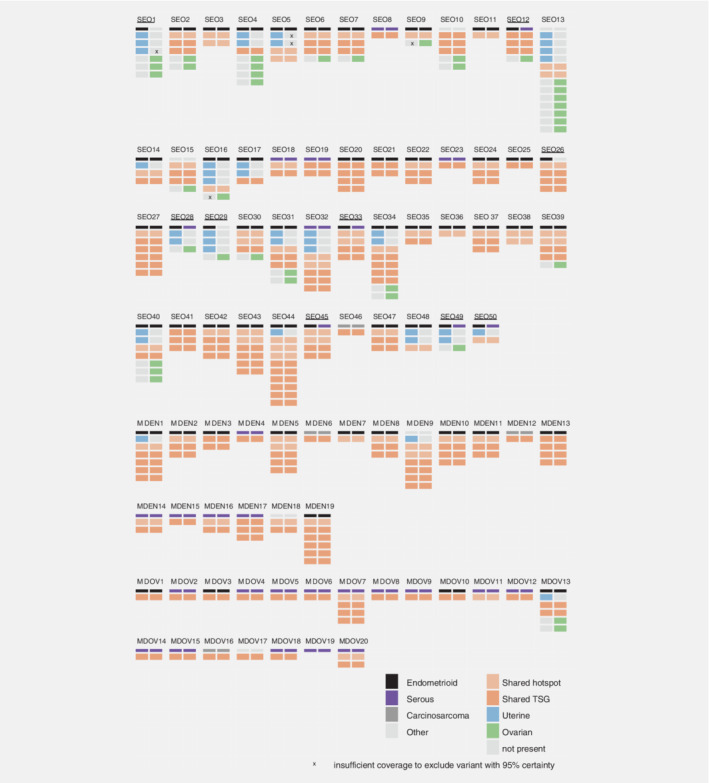
Display of all next‐generation sequencing derived somatic mutations detected in all cases, diagnosed as either synchronous endometrial and ovarian carcinoma (SEO), endometrial carcinoma with ovarian metastasis (MDEN) or ovarian carcinoma with endometrial metastasis (MDOV). For each case, mutations present in the endometrial carcinoma are presented in the left column, and mutations present in the ovarian carcinoma are presented in the right column. Shared mutations are shown in orange, mutations only present in the endometrial carcinoma are shown in blue, and mutations only present in the ovarian carcinoma are shown in green. In case a mutation was not found in the corresponding carcinoma, a cross indicates that the coverage on a specific locus was not sufficient to exclude the mutation with 95%‐certainty. Abbreviation: TSG, tumor suppressor gene.

A total of 109 patients were identified, of which 20 patients were excluded, because of the absence of tumor tissue (*n* = 9) or technical failure of the sequencing (*n* = 11), leaving 89 patients for analysis. After histopathological review, 50 cases (56.2%) were diagnosed as SEOs according to the Scully criteria; 19 cases (21.3%) as endometrial MD; 20 cases (22.5%) as ovarian MD. Of patients with SEOs, 30.0% (15/50) had endometriosis, compared to 10.5% (2/19) of patients with endometrial MD and 5.0% (1/20) of patients with ovarian MD (Table 1). In total, 56.0% (28/50) of patients with SEOs received adjuvant chemotherapy, compared to 42.1% (8/19) of patients with endometrial MD and 85.0% (17/20) of patients with ovarian MD. Ten per cent (5/50) of patients with SEOs received adjuvant radiotherapy, compared to 31.6 and 0%, respectively. Uterine histology was endometrioid in 82.0% of SEOs (41/50), 52.6% of endometrial MD cases (10/19) and 20.0% of ovarian MD cases (4/20). In SEOs, concordant histology between endometrial and ovarian carcinoma was present in 82.0% (41/50, Fig. 1).

**Table 1:** Baseline characteristics of included patients

	SEO (*n* = 50)	Endometrial MD (*n* = 19)	Ovarian MD (*n* = 20)
Age (years)	56 (31–82)^1^^,^ 2	67 (43–88)^1^	65 (50–78)^2^
BMI (kg/m^2^)	28 (20–48)	31 (18–44)	28 (23–31)
Ca‐125 level at diagnosis (IU/ml)	210 (4–14.500)	763 (133–6.553)	562 (5–12.039)
Follow‐up (months)	49 (0–214)^1^^,^ 2	11 (0–61)^1^	24 (0–60)^2^
Menopausal state			
Premenopausal	19 (38.0)^1^^,^ 2	2 (10.5)^1^	0 (0)^2^
Postmenopausal	26 (52.0)	16 (84.2)	19 (95.0)
Unknown	5 (10.0)	1 (5.3)	1 (5.0)
Endometriosis present			
Yes	15 (30.0)^2^	2 (10.5)	1 (5.0)^2^
No	35 (70.0)	17 (89.5)	19 (95.0)
Histology EC			
Endometrioid	41 (82)^1^^,^ 2	10 (52.6)^1^	4 (20.0)^2^
Serous	5 (10)	5 (26.3)	14 (70.0)
Carcinosarcoma	1 (2)	2 (10.5)	1 (5.0)
Other	3 (6)	2 (10.5)	1 (5.0)
Grade EC			
1	24 (48.0)^1^^,^ 2	2 (10.5)^1^^,^ 3	0 (0)^2^^,^ 3
2	18 (36.0)	7 (36.8)	2 (10.0)
3	8 (16.0)	10 (52.6)	18 (90.0)
Uterine FIGO stage			
I	37 (74.0)^1^	0 (0)^1^	
II	8 (16.0)	0 (0)	
IIIA	2 (4.0)	9 (47.4)	
IIIB	0 (0)	0 (0)	
IIIC	2 (4.0)	5 (26.3)	
IV	1 (2.0)	5 (26.3)	
Histology OC			
Endometrioid	32 (62.0)^2^	10 (52.6)	4 (20.0)^2^
Serous	11 (22.0)	5 (26.3)	14 (70.0)
Carcinosarcoma	1 (2.0)	2 (10.5)	1 (5.0)
Other	6 (12.0)	2 (10.5)	1 (5.0)
Grade OC			
1	13 (26.0)^2^	2 (10.5)^3^	0 (0)^2^^,^ 3
2	18 (36.0)	7 (36.8)	1 (5.0)
3	19 (38.0)	10 (52.6)	19 (95.0)
Ovarian FIGO stage			
I	25 (50.0)^2^		0 (0)^2^
II	11 (22.0)		1 (5.0)
IIIA	3 (6.0)		0 (0)
IIIB	0 (0)		0 (0)
IIIC	10 (20.0)		16 (80.0)
IV	1 (2.0)		3 (15.0)
Ovaries bilaterally involved			
No			
Yes	37 (74.0)	13 (68.4)	14 (70.0)
	13 (26.0)	6 (31.6)	6 (30.0)
Adjuvant therapy			
None	14 (28.0)	3 (15.8)^3^	3 (15.0)^3^
Radiotherapy	5 (10.0)	6 (31.6)	0 (0)
Chemotherapy	28 (56.0)	8 (42.1)	17 (85.0)
Chemoradiation	3 (6.0)	0 (0)	0 (0)
Unknown		2 (10.5)	

*p* values were obtained using the Fisher's exact test and χ^2^. Values are presented as median (range) or number (%).

Abbreviations: BMI, body mass index; Ca‐125, cancer antigen 125; EC, endometrial carcinoma; MD, metastatic disease; OC, ovarian carcinoma; SEO, synchronous endometrial and ovarian cancer.

1 *p* < 0.05 comparing SEO with endometrial MD.

2 *p* < 0.05 comparing SEO with ovarian MD.

3 *p* < 0.05 comparing endometrial MD with ovarian MD.

### Shared mutations

At least 70 independent gDNA molecules were sequenced in 78% of all sequenced exons, which was sufficient to exclude variants with a 10% VAF. In 92.0% of SEOs (46/50), the endometrial and concurrent ovarian carcinoma had at least one somatic mutation in common (Fig. 1, Supporting Information Table S3). Of these, 12 shared one mutation including six cases with an “activating hotspot” mutation (50.0%), and six cases with a tumor suppressor gene mutation in either *ARID1A*, *PTEN* or *TP53* (50.0%). As can be seen in Figure 1, 23 (56.0%) had two or three mutations in common, eight (16.0%) shared four or five mutations, and three (6.0%) shared six or more mutations. In all 46 cases having one or more mutations in common, the clonality indices (CIs) were above 0.95, indicating that it is unlikely these mutations co‐occurred by coincidence (Supporting Information Fig. S1). The CI was above 0.95 even for all six cases that only shared one activating hotspot mutation in *KRAS*, *CTNNB1* or *PIK3CA*, that are known to be recurrent in EC. To illustrate, the most prevalent hotspot mutation in our dataset (PIK3CA:c.3140A>G(p.(His1047Arg))) was found in 12 of 240 cases included in the TCGA public dataset (expected frequency: 5%).

In all patients with MD, the endometrial and concurrent metastasis had at least one somatic mutation in common, except for one ovarian MD case in which no mutation was found (case 19, Fig. 1). Of these, 53.8% (21/39) had one mutation in common, with a maximum of six shared mutations in two MD cases (MDEN9, MDEN19). The CIs for all MD cases were above 0.95.

### Unique mutations

As can be appreciated in Figure 1, several cases harbored unique mutations in addition to the shared mutations in either the ovarian or the endometrial carcinoma, an observation seemingly specific to the SEOs. In total, 34% of SEOs (17/50) harbored at least one unique mutation only present in the endometrial carcinoma, compared to 10.5% in the endometrial MD cases (2/19) and 5.0% in the ovarian MD cases (1/20). *Vice versa*, 38% of SEOs (19/50) harbored at least one mutation only present in the ovarian carcinoma, compared to 0% in the endometrial MD cases, and 5.0% in the ovarian MD cases (1/20).

To explore whether the presence of these unique mutations could be explained by subclonal mutations due to tumor heterogeneity, these unique mutations were compared to regard to variant allele frequency (VAF), which reflects the frequency of the mutant alleles compared to the total amount of alleles sequenced. In this perspective, low VAFs could indicate the presence of subclonal mutations and tumor heterogeneity. Interestingly, the variant allele frequencies (VAFs) of unique mutations were not significantly lower than the VAFs of shared mutations in the same specimens, suggesting tumor heterogeneity within the tested lesions is not a likely explanation for the presence of unique mutations in the SEOs (Supporting Information Fig. S2). This was confirmed in analysis for oncogenes and TSGs separately (Supporting Information Fig. S3). Pathogenic mutations are considered to accumulate over time, with mutations in particular genes described as “early” or “late” events. In the SEOs, especially mutations in *ARID1A* (35.0%), followed by *PTEN*, were found to be unique, suggesting to originate from “late” events (Supporting Information Table S4).

### Concordance of histology and mutation pattern

All 41 SEOS with concordant histology in both carcinomas shared at least one mutation supporting a clonal origin. Nine SEOs were diagnosed with “discordant” histology, for example, endometrioid histology in the endometrial carcinoma and nonendometrioid histology in the ovarian carcinoma. Four of these SEOs had no mutations in common, suggesting a nonclonal origin, whereas molecular analysis suggested clonal origin for the other five cases (Table 2).

**Table 2:** Characteristics of nine SEO cases with discordant histology

	Concordance index = 0				Concordance index >0.95				
	SEO1	SEO28	SEO29	SEO49	SEO12	SEO26	SEO33	SEO45	SEO50
Age	65	79	69	73	76	74	43	56	82
Ca‐125 (IU/ml)	14.8	98	1,200	97	489	−	19	251	1,111
Histology endometrium	EEC	EEC	EEC	EEC	EEC	EEC	EEC	EEC	EEC
Grade endometrium	1	1	1	2	2	2	1	1	2
MI	<50%	<50%	>50%	<50%	>50%	>50%	No	<50%	<50%
Endometrial hyperplasia	+	+	+	+	−	+	+	−	+
Mutations endometrium	*ARID1A* 1 *PTEN*	*PTEN TP53* 1	*CTNNB1 PTEN*	*ARID1A PTEN*	*ARID1A* *PTEN*	*ARID1A KRAS PTEN*	*ARID1A KRAS* *PIK3CA PTEN*	*ARID1A KRAS PIK3CA PTEN*	*KRAS PIK3CA*
Histology ovary	Clear cell	Serous	Clear cell	Serous	Serous	Clear cell	Serous	Serous	Serous
Grade ovary	3	3	3	3	3	3	1	3	3
Mutations ovary	*ARID1A* 1 *PIK3CA*	*TP53* 1	*TP53*	*PIK3CA*	*ARID1A* *PTEN* *TP53*	*ARID1A KRAS PTEN*	*ARID1A KRAS* *PIK3CA PTEN*	*ARID1A KRAS PIK3CA PTEN*	*PIK3CA*
Recurrence	−	+	+	−	+	−	−	−	+
Death	−	+	+	−	+	−	−	−	+

Abbreviations: EEC, endometrioid endometrial carcinoma; SEO, synchronous endometrial and ovarian carcinoma; Ca‐125, cancer antigen 125; MI, myometrial invasion.

1 Nonidentical mutations.

### Molecular profile per subgroup

**Figure 2:**
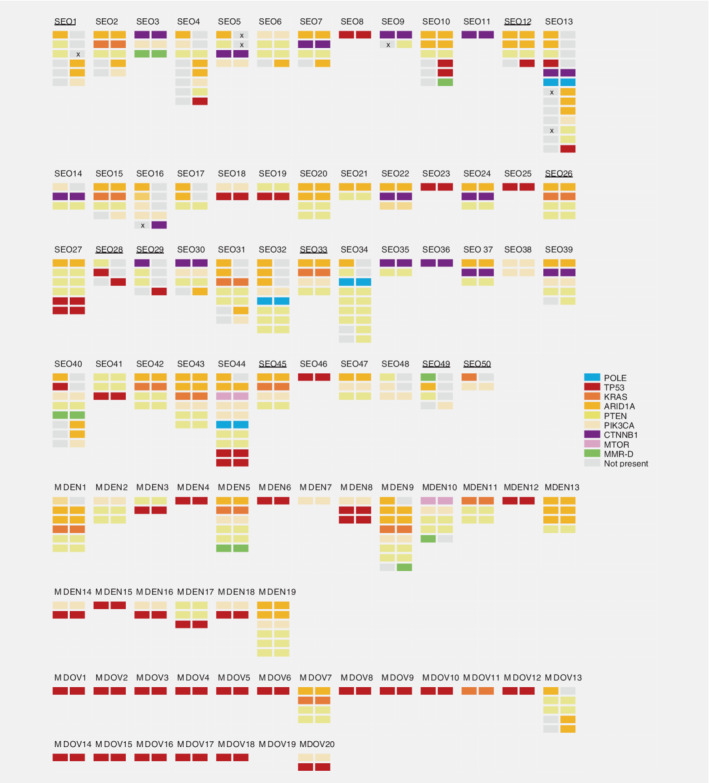
Display of all next‐generation sequencing derived mutated genes detected in all cases, diagnosed as either synchronous endometrial and ovarian carcinoma (SEO), endometrial carcinoma with ovarian metastasis (MDEN) or ovarian carcinoma with endometrial metastasis (MDOV). The colors indicate specific genes (see legend).

Molecular profiles between the three subgroups were compared, stratified by histological subtype. First, the three subgroups were compared analyzing with only those with endometrioid histology in both counterparts. For these analyses, only mutations present in both counterparts were considered for each tumor pair. Although numbers were limited, we found that *CTNNB1* was mutated significantly more frequent in SEOs than in endometrial MD cases (40.6% *vs*. 0%, *p* = 0.018, Fig. 2, Supporting Information Table S5). Also, we found that *TP53* was mutated less frequently in SEOs than in ovarian MD cases (12.5% *vs*. 75.0%, *p* = 0.018). Analyzing only carcinomas with nonendometrioid histology, we found that *PTEN* was mutated significantly more frequent in SEOs than in ovarian MD cases (44.4% *vs*. 6.3%, *p* = 0.019). Interestingly, *TP53* mutations were found less frequent in SEOs than in endometrial MD cases (27.8% *vs*. 88.9%, *p* = 0.004) and ovarian MD cases (27.8% *vs*. 81.3%, *p* = 0.003). It should be noted that in eight SEOs classified as nonendometrioid, also a component of endometrioid histology was found (Fig. 2), which may partly explain the differences in mutational profiles. Loss of one of the MMR proteins in both the endometrial and ovarian carcinoma was seen in 4.0% of SEOs (2/50), compared to 5.3% of endometrial MD cases (1/19) and 0% of ovarian MD cases (Fig. 2).

### TCGA analysis supports a primary endometrial origin for SEOs

**Figure 3:**
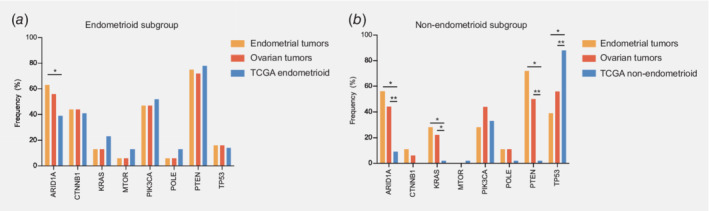
Mutational profiles from the publicly available TCGA endometrial carcinoma tumor set was compared to the mutational profiles from the synchronous endometrial and ovarian cancers. Both groups were stratified by histological subtype (endometrioid, nonendometrioid). (*a*) The endometrioid tumors in the study cohort (*n* = 32) were compared to the endometrioid tumors from the publicly available TCGA dataset (*n* = 193). From each tumor pair, both the endometrial and ovarian counterpart were compared. (*b*) The nonendometrioid tumors in the study cohort (*n* = 18) were compared to the nonendometrioid tumors from the TCGA dataset (*n* = 43). From each tumor pair, both the endometrial and ovarian counterpart were compared. Abbreviation: TCGA, The Cancer Genome Atlas. *p < 0.05; **p < 0.005. [Color figure can be viewed at wileyonlinelibrary.com]

The TCGA 2013 endometrial carcinoma tumor set was used to compare the molecular profiles with those from the SEO subgroup (Fig. 3). To this extent, we stratified for histological subtype (endometrioid and nonendometrioid), and compared both the endometrial and ovarian counterpart from each tumor pair. The molecular profiles obtained from the TCGA 2013 endometrioid endometrial carcinoma tumor set (*n* = 193) were similar to the endometrioid SEO molecular profiles (*n* = 32) for both the endometrial and ovarian counterparts, except for a higher frequency of *ARID1A* mutations in the endometrial tumors. Comparing the nonendometrioid SEOs (*n* = 18) with the TCGA 2013 serous endometrial carcinoma tumor set (*n* = 43), molecular profiles were similar except for *ARID1A*, *KRAS*, *PTEN* and *TP53*. Similar analyses, including only mutations present in both components of each tumor pair, are shown in Supporting Information Table S6.

### Outcome related to molecular profile

**Figure 4:**
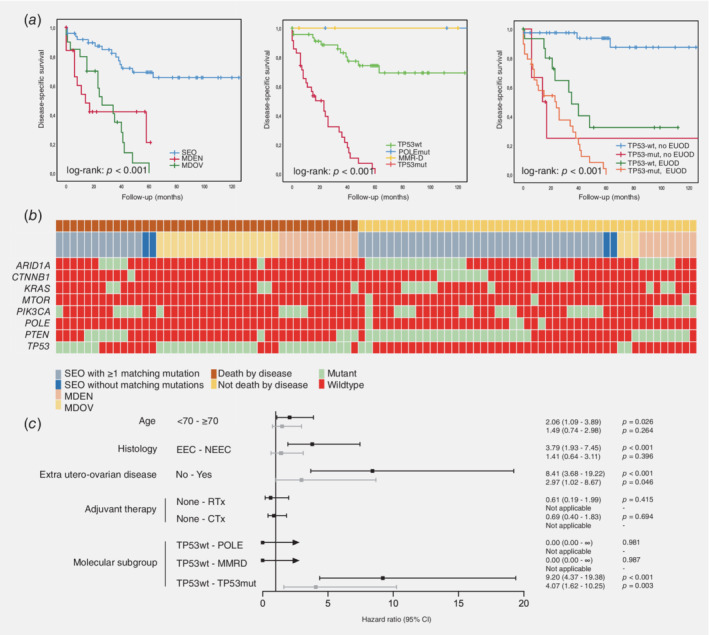
(*a*) Kaplan–Meier curves displaying disease‐specific survival in cases according to molecular classification classifying four subgroups (*POLE* mutated, MMR‐D, *TP53* wild‐type and *TP53* mutant based on sequencing analysis, left panel); by diagnosis according to the Scully criteria (middle panel), by *TP53*‐status and the presence of extra utero‐ovarian disease (right panel). (*b*) Molecular characteristics grouped by outcome. Only mutations that were present in both the endometrial and corresponding ovarian carcinoma were included in the figure. (*c*) Univariable and multivariable Cox regression analysis. The hazard ratios with 95% confidence intervals are depicted by the black lines. All risk factors significantly associated (*p* < 0.10) with disease‐specific survival in univariable analysis were included in the multivariable Cox regression analysis, depicted by the gray lines. For *POLE* and MMR‐D, no confidence intervals could be calculated, because no events were observed in these subgroups. Abbreviations: CTx, chemotherapy; EC, endometrial carcinoma; EEC, endometrioid endometrial carcinoma; EUOD, extra utero‐ovarian disease; MDEN, endometrial carcinoma with ovarian metastasis; MDOV, ovarian carcinoma with endometrial metastasis; MMR‐D, mismatch repair deficient; NEEC, nonendometrioid endometrial carcinoma; RTx, radiotherapy; SEO, synchronous endometrial and ovarian carcinoma; TP53wt, *TP53* wildtype; TP53mut, *TP53* mutant.

The 5‐year DSS was better for SEOs than for endometrial MD cases and ovarian MD cases (log‐rank: *p* < 0.001 for both, Fig. 4 *a* left panel). Subsequently, outcome was correlated to molecular profile using only mutations shared between both corresponding carcinomas as these would represent true tumor‐driving mutations. Classification according to the ProMisE subgroups identified four groups with separate survival curves, with *TP53*‐mutated group having the worst disease‐specific survival (log‐rank: *p* < 0.001, Fig. 4 *a* middle panel).

Figure 4 *b* shows the molecular characteristics stratified by outcome. In those who died because of the disease, *TP53* mutations were seen more frequently (71.4% *vs*. 10.6%, *p* < 0.001). Less frequently seen were mutations in *CTNNB1* (2.4% *vs*. 27.7%, *p* < 0.001), *PTEN* (23.8% *vs*. 68.1%, *p* < 0.001) and *ARID1A* (11.9% *vs*. 44.7%, p < 0.001).

Multivariable Cox regression analysis showed that molecular subgroup (*TP53* mutant), and the presence of extra‐utero‐ovarian disease were independent predictors for poor clinical outcome (Fig. 4 *c*). Combining these two risk factors, survival can be extracted from the Kaplan–Meier curve in Figure 4 *a* (right). Supporting Information Figure S4 included a summarizing figure of the results.

## Discussion

In our study, we have shown that SEOs, classified according to the Scully criteria, are clonally related in an overwhelming majority of cases. In total, 92% of SEOs shared one or more somatic mutations with high clonality indices supporting clonal origin. Discordant histology indicated a nonclonal origin in half of these cases. SEOs were enriched for *PTEN* and *CTNNB1* mutations and harbored less *TP53* mutations than MD cases. There were striking similarities between the molecular profiles from the SEO subgroup and the TCGA 2013 endometrial carcinoma tumor set, implying the endometrium could be the primary origin for these cases rather than the ovary. *TP53* mutations and the presence of extra‐utero‐ovarian disease were associated with poor outcome. Despite the fact that the majority of patients with *TP53* mutated carcinomas received chemotherapy, clinical outcome was poor, underlining the need for more effective (targeted) therapies in this subgroup.

This is the largest series so far, confirming the clonal relationship between both carcinomas in concurrent cases diagnosed as SEOs, implying these carcinomas actually represent (pseudo)metastatic disease, rather than two independently evolved carcinomas.^10^, ^11^, ^12^, ^14^ The mechanisms underlying the spread from one anatomic site to another, without carrying a dismal prognosis are not fully understood. One possible explanation is that these cells are not able to actually invade the circulation and spread to distant sites, but detach and spread to nearby sites as the ovary, rather representing “pseudometastasis” than actual wide‐spread metastatic disease. In the current study, this was supported by a low occurrence of lymphovascular space invasion in SEOs (17%) compared to MD (78%). Endometriosis was present more often in SEOs (30.0%) than in MD (10.5 and 5.0%), which implies the Müllerian tract in these patients may be more subjected to retrograde flux supporting local “pseudometastasis” of cancer cells.

By comparing the molecular profiles from these SEOs with those from the TCGA endometrial carcinoma tumor set, we concluded that the endometrium could be designated as the primary origin.^15^, ^22^ The profiles of the endometrioid subgroups were very similar between our study and the TCGA, whereas the profiles of the nonendometrioid subgroups differed with respect to the presence of *ARID1A*, *KRAS*, *PTEN* and *TP53* mutations. Yet, our cohort was enriched for mixed carcinomas, whereas the TCGA dataset only comprised serous carcinomas, which might explain these differences. In the endometrioid SEO subgroup, we found *PTEN* mutations in 72% (ovarian counterpart)–75% (endometrial counterpart), which was similar to the mutation rate found in endometrioid endometrial carcinomas by McConechy *et al*. (67%).^24^ In contrast, they showed that *PTEN* mutations are less common in endometrioid ovarian carcinomas, found in only 17%. Our findings imply that most SEOs rather represent a subgroup of (pseudo)metastatic endometrial cancers presenting with indolent behavior and good clinical prognosis.

Interestingly, four SEOs (8%) shared no mutations, in the presence of multiple unique mutations, which suggests that these cases truly represent independent carcinomas rather than metastatic disease. In these cases, the endometrial carcinoma was of low‐grade endometrioid histology, whereas the ovarian carcinoma was of high‐grade nonendometrioid histology. In contrast, all cases with concordant histology between endometrial and ovarian carcinoma were considered clonally related based on molecular profile, implying that concordant histology can be seen as a strong histopathological argument favoring a clonal origin.

Although some mutations in SEOs are shared between the endometrial and ovarian tumor, other mutations are unique for one of the tumors. The increased prevalence of unique mutations in the SEO subgroup can be indicative of an early shared precursor, followed by independent tumorigenesis and local outgrowth, explaining these genetic divergent profiles. Especially mutations in *ARID1A* were found to be unique frequently (35%), suggesting these mutations occur often as a “late” event and are context‐dependent, possibly secondary to, for example, *POLE* mutations or MMR‐D. This observation is in line with other studies in EC as well as hepatocellular carcinoma.^25^, ^26^

We have shown that SEOs harbored a profoundly different molecular profile compared to metastatic disease, with more frequently *PTEN* and *CTNNB1* mutations and less frequently *TP53* mutations. Although earlier studies did not directly compare molecular profiles of SEOs and MD, Chao *et al*. found in a series of 14 SEOs frequent *ARID1A* and *CTNNB1* mutations as well.^12^ Moreover, a high frequency of *CTNNB1* mutations was found by Ishikawa *et al*. in a series of eight SEOs.^13^

Classification with the ProMisE criteria revealed that *TP53* mutations were independently associated with a poor outcome, after adjusting for co‐variates. Earlier studies pointed out that adjuvant treatment planning in these SEOs should not be altered based on the finding alone that they were actually clonally related, because of their generally favorable outcome.^10^, ^27^ The favorable prognosis is supported in the current study, and we have identified molecular profile and the presence of extra‐utero‐ovarian disease as predictors that may in the future be used to guide adjuvant treatment planning, irrespective of the histopathological classification. The clinical outcome of patients with *TP53* wild‐type disease confined to the uterus and ovary was excellent. Although prospective evaluation should confirm these results, adjuvant treatment may be omitted in these specific cases. Given the increased risk of metastatic disease and poor clinical outcome, systematic therapy could be indicated such as chemotherapy or targeted agents. Even though not significant, *POLE* mutated and MMR‐D carcinomas had a favorable outcome, in line with earlier studies.^19^, ^20^, ^21^

We have investigated a large series of concurrent endometrial and ovarian carcinomas, histopathologically reviewed by two expert pathologists. We have compared the molecular profile of cases classified as SEOs with those of cases with MD. Yet, there are some limitations that need to be addressed. The clonal relationship of the four SEOs in which no mutations were found remains unclear. As our NGS panel only targets a small proportion of the entire genome, genetic analysis beyond our gene panel could be able to clarify these cases.

Concluding, the current study has shown that SEOs are clonally related in an overwhelming majority of cases, with a favorable prognosis and a molecular profile suggesting the endometrium as primary origin. *TP53* mutation and extra‐utero‐ovarian disease were independent predictors of poor outcome. Therefore, assessment of *TP53* mutational status by either NGS or immunohistochemistry is recommended in order to risk‐stratify these patients for systemic adjuvant treatment.

## Conflict of interest

The authors declare no potential conflicts of interest.

## Supporting information


**Appendix S1** Supporting information
