# Supplementary material for: Molecular profiling identifies synchronous endometrial and ovarian cancers as metastatic endometrial cancer with favorable clinical outcome
Source: Int J Cancer. 2020 Feb 18;147(2):478–89. doi: 10.1002/ijc.32907 (PMC7317735; doi:10.1002/ijc.32907)
Supplement: Supplementary file 1 — Appendix S1 Supporting information [file IJC-147-478-s001.pdf]

Supplementary material

Molecular profiling identifies synchronous endometrial and ovarian cancers as metastatic endometrial cancer with favorable clinical outcome

C. Reijnen, H.V.N. Küsters-Vandevelde MD, PhD, M.J.L. Ligtenberg, PhD, J. Bulten MD, PhD, M. Oosterwegel, M.P.L.M. Snijders MD, PhD, S. Sweegers, J.A. de Hullu MD, PhD, M.C. Vos MD, A.A.M. van der Wurff MD, PhD, A.M. van Altena MD, PhD, A. Eijkelenboom, PhD, J.M.A. Pijnenborg MD, PhD

Supplementary Figure 1. Clonality indices for all cases, based on (A.) mutation frequencies obtained from the online The Cancer Genome Atlas (TCGA) endometrial carcinoma database, or (B.) mutation frequencies obtained from the online TCGA ovarian carcinoma database.
Supplementary Figure 2. Variant allele frequencies (VAFs) of shared vs. unique mutations.
Supplementary Figure 3. Normalized variant allele frequencies (VAFs) of shared and unique oncogenes and tumor suppressor genes (TSGs), respectively. Normalized VAGs were calculated by dividing the mean VAF of either the shared or unique mutations in either TSGs or oncogenes, with the mean VAF of all mutations in either TSGs or oncogenes in the specific specimen.
Supplementary Figure 4. Summarizing figure of the results.
Supplementary Table 1. Sculley criteria.
Supplementary Table 2. All gene regions targeted by the smMIP panel.
Supplementary Table 3. (potentially) pathogenic variants in all cases.
Supplementary Table 4. Frequency of cases harboring a shared vs. unique mutation, specified for each gene.
Supplementary Table 5. Variants found per subgroup.
Supplementary Table 6. The Cancer Genome Atlas analysis to compare mutational profiles from the publicly available TCGA endometrial carcinoma tumor set with synchronous endometrial and ovarian cancers.

Supplementary Figure 1. Clonality indices for all cases, based on (A.) mutation frequencies obtained from the online The Cancer Genome Atlas (TCGA) endometrial carcinoma database, or (B.) mutation frequencies obtained from the online TCGA ovarian carcinoma database.

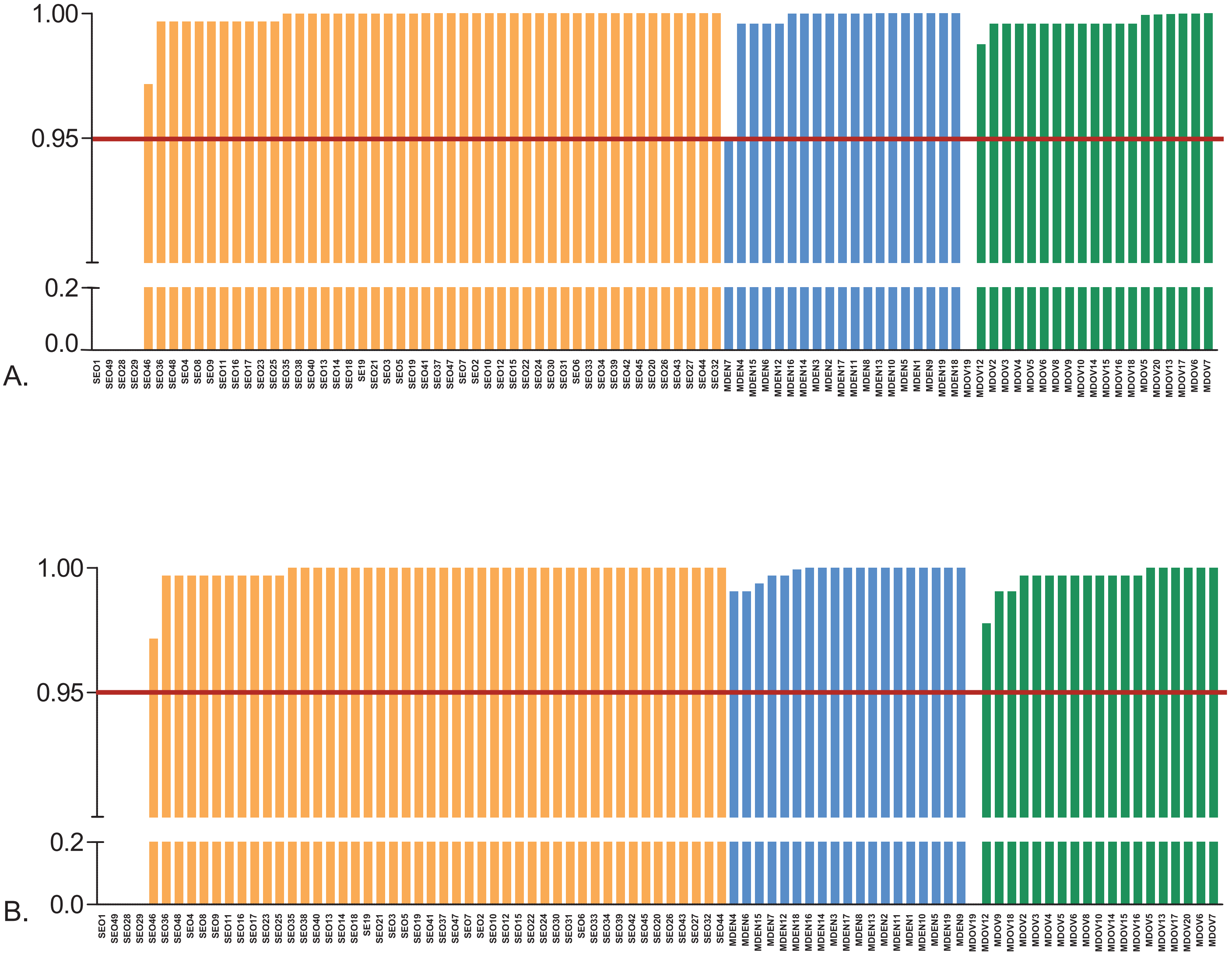

Supplementary Figure 2. Variant allele frequencies (VAFs) of shared vs. unique mutations.

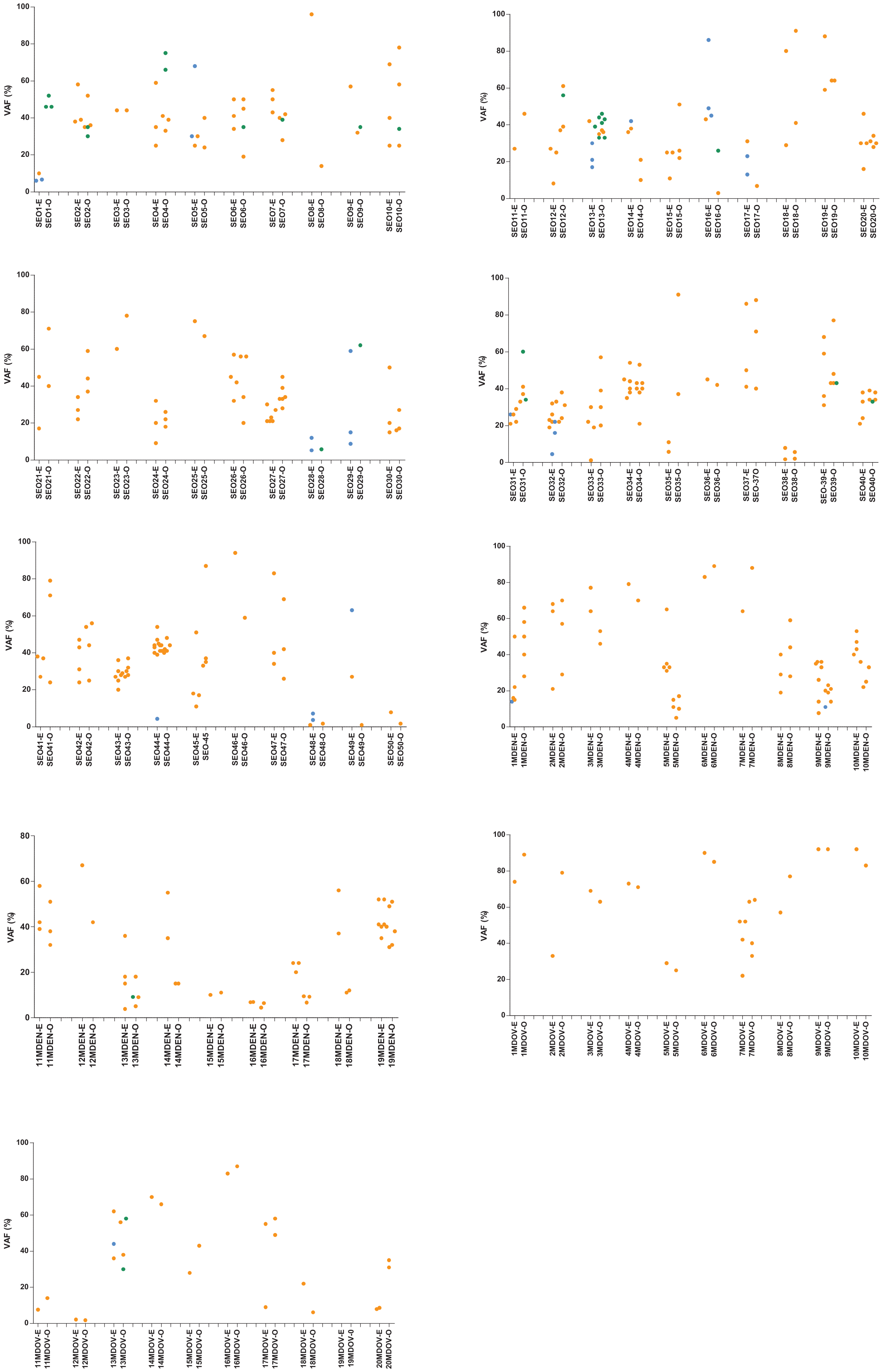

Supplementary Figure 3. Normalized variant allele frequencies (VAFs) of shared and unique oncogenes and tumor suppressor genes (TSGs), respectively. Normalized VAFs were calculated by dividing the mean VAF of either the shared or unique mutations in either TSGs or oncogenes, with the mean VAF of all mutations in either TSGs or oncogenes in the specific specimen.

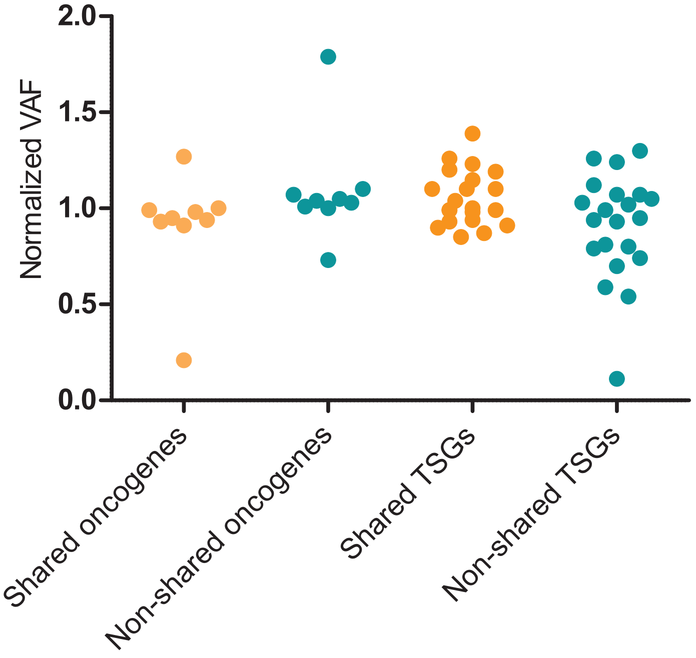

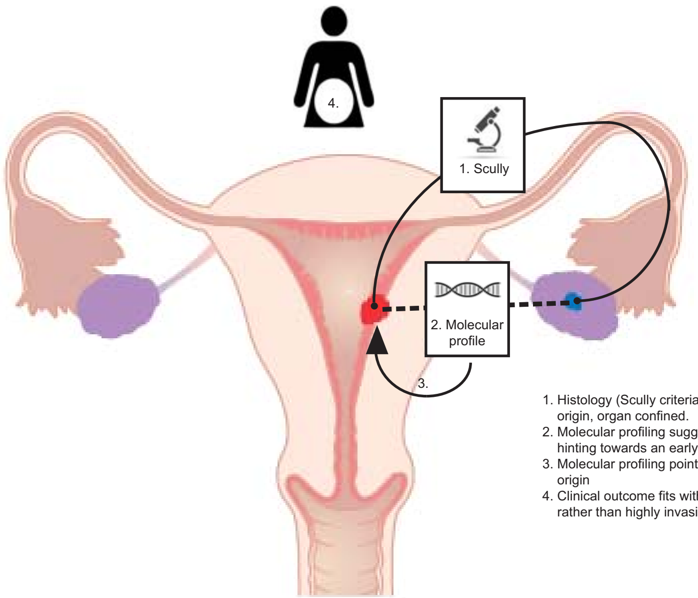

1. Histology (Scully criteria) suggests independent origin, organ confined.
2. Molecular profiling suggests common origin, hinting towards an early shared precursor.
3. Molecular profiling points towards endometrial origin
4. Clinical outcome fits with locally spread disease, rather than highly invasive carcinoma

Supplementary Figure 4: Summarizing figure of the results

Supplementary Table 1. Sculley criteria

	Primary Endometrial Cancer with ovarian metastases	Primary Ovarian Cancer with endometrial metastases	Independent Primary Tumors of Endometrium and Ovary
1	Histologic similarity of the tumors	Histologic similarity of the tumors	Histologic dissimilarity of the tumors
2	Large endometrial tumor and small ovarian tumor(s)	Large ovarian tumor and small endometrial tumor(s)	No or only superficial myometrial invasion of endometrial tumor
3	Atypical endometrial hyperplasia additionally present	Ovarian endometriosis present	No vascular space invasion of endometrial tumor
4	Deep myometrial invasion<br>a. Direct extension into the adnexa<br>b. Vascular space invasion in myometrium	Location in ovarian parenchyma	Atypical endometrial hyperplasia additionally present
	Spread elsewhere in typical pattern of endometrial carcinoma	Direct extension from ovary predominantly into outer wall of uterus	Absence of other evidence of spread of endometrial tumor
6	Ovarian tumors bilateral and/or multinodular	Spread elsewhere in typical pattern of ovarian carcinoma	Ovarian tumor unilateral (80 to 90 percent of cases)
7	Hilar location, vascular space invasion, surface implants, or combination in ovary	Ovarian tumor unilateral (80 to 90 percent of cases) and forming single mass	Ovarian tumor located in parenchyma
8	Ovarian endometriosis absent	No atypical endometrial hyperplasia	No vascular space invasion, surface implants, or predominant hilar location in ovary
9	Aneuploidy with similar DNA indices or diploidy of both tumors[a]	Aneuploidy with similar DNA indices or diploidy of both tumors[a]	Absence of other evidence of spread of ovarian tumor
10	Similar molecular genetic or karyotypic abnormalities in both tumors	Similar molecular genetic or karyotypic abnormalities in both tumors	Ovarian endometriosis present
11			Different ploidy of DNA indices, if aneuploid, of the tumors[a]
12			Dissimilar molecular genetic or karyotypical abnormalities in the tumors

[a]The possibility of tumor heterogeneity must be taken into account in the evaluation of ploidy findings.

Supplementary Table 2. All gene regions targeted by the smMIP panel

Gene	Exon	Targeted codons	Positions	RefSeq ID	Ensembl ID
ARID1A	01 to 20	M1-Stop2286	c.1 to c.6858	NM_006015.5	ENST00000324856
CTNNB1	3	D32-S45	c.53 to c.146	NM_001904.3	ENST00000349496
KRAS	2	G12-G13	c.9 to c.71	NM_004985.4	ENST00000311936
	3	A59-Q61	c.122 to c.215		
	4	K117, A146	c.291-5 to c.357		
			c.402 to c.450+5		
MTOR	30	D1458-E1489	c.4371 to c.4469+5	NM_004958.3	ENST00000361445
	39	A1789-A1820	c.5365-5 to c.5460		
	43	A1971-L1995	c.5911-5 to c.5985		
	47	Q2194-L2220	c.6580 to c.6662+5		
	53	M2404-D2433	c.7210 to c.7300+5		
	56	G2484-T2509	c.7448-5 to c.7527		
PIK3CA	2	S66 – I117	c.195 to c.352	NM_006218.3	ENST00000263967
	5	Y317 – K353	c.947 to c.1059		
	8	E418 – M441	c.1252 to c.1323		
	10	D520 – H554	c.1558 to c.1664		
	21	S1015 – N1068	c.3058 to c.3207		
POLE	9 to 14	D268-E491	c.802-5 to c.1473+5	NM_006231.3	ENST00000320574
PTEN	1 to 9	M1-Stop404	c.1 to c.1210+5	NM_000314.6	ENST00000371953
TP53	2 to 11	>95% of all coding and splice sequences (-5/+5)	c.1 to c.1180+5	NM_000565.5	ENST00000269305

Supplementary Table 3. (potentially) pathogenic variants in all cases

		Endometrial carcinoma			Ovarian carcinoma		
Patient ID	Variant	TCP	VAF	Mutant coverage	TCP	VAF	Mutant coverage
SEO1	ARID1A:c.5734G>A(p.(Asp1912Asn))[a]	20%	10%	100	40%	NA	250
	PTEN:c.44_56(p.(Arg15fs))		6%	400		NA	484
	PTEN:c.890delA(p.(Asp297fs))		7%	284		NA	392
	ARID1A:c.267_286del(p.(Ser90fs))		NA	250		47%	289
	ARID1A:c.6611_6621del(p.(Ala2204fs))		NA	90		46%	52
	PIK3CA:c.1633G>A(p.(Glu545Lys))		NA	322		52%	438
SEO2	ARID1A:c.1534C>T(p.(Gln512*))	60%	NA	2261	60%	35%	423
	ARID1A:c.3598C>T(p.(Gln1200*))		39%	2223		36%	539
	KRAS:c.35G>T(p.(Gly12Val))		58%	3200		52%	342
	PIK3CA:c.263G>A(p.(Arg88Gln))		NA	1118		20%	100
	PTEN:c.70G>C(p.(Asp24His))		38%	6347		35%	1440
SEO3	CTNNB1:c.98C>G(p.(Ser33Cys))	80%	47%	249	80%	35%	86
	PIK3CA:c.3140A>G(p.(His1047Arg))		44%	155		42%	33
SEO4	ARID1A:c.618_619dup(p.(Phe207fs))	70%	36%	44	50%	NA	186
	ARID1A:c.1338_1339del(p.(Gln449fs))		NA	138		41%	112
	ARID1A:c.6046delC(p.(Leu2016fs))		NA	387		39%	344
	PIK3CA:c.278G>A(p.(Arg93Gln))		35%	46		NA	74
	PIK3CA:c.1624G>A(p.(Glu542Lys))		25%	328		2%	316
	PIK3CA:c.1633G>A(p.(Glu545Lys))		NA	40		33%	321
	PTEN:c.891delT(p.(Gln298fs))		59%	154		66%	294
	TP53:c.647T>A(p.(Val216Glu))		NA	249		75%	356
SEO5	ARID1A:c.326dupC(p.(Arg110*))	70%	68%	132	60%	NA	26
	CTNNB1:c.109T>G(p.(Ser37Ala))		25%	56		24%	33
	PIK3CA:c.3140A>G(p.(His1047Arg))		30%	47		40%	10
	PTEN:c.389G>A(p.(Arg130Gln))		30%	60		NA	16
SEO6	ARID1A:c.1159_1162dup(p.(Gly388fs))	50%	NA	64	60%	20%	175

	PIK3CA:c.3140A>G(p.(His1047Arg))		34%	88		28%	71
	PTEN:c.89C>T(p.(Pro30Leu))[a]		50%	32		38%	32
	PTEN:c.955_958del(p.(Thr319*))		41%	141		53%	143
SEO7	ARID1A:c.1489C>T(p.(Gln497*))	60%	50%	28	40%	28%	161
	ARID1A:c.6703_6704del(p.(Ala2235fs))		NA	82		40%	30
	CTNNB1:c.110C>T(p.(Ser37Phe))		43%	14		42%	24
	PTEN:c.388C>G(p.(Arg130Gly))		55%	22		39%	26
SEO8	TP53:c.713G>A(p.(Cys238Tyr))	80%	86%	44	80%	57%	7
SEO9	CTNNB1:c.110C>T(p.(Ser37Phe))	60%	57%	28	60%	32%	94
	PTEN:c.412T>G(p.(Tyr138Asp))[a]		NA	10		31%	77
SEO10	ARID1A:c.4286(p.(Asn1429fs))	60%	25%	56	60%	25%	264
	ARID1A:c.4840C>T(p.(Gln1614*))		40%	40		58%	203
	PTEN:c.70G>T(p.(Asp24Tyr))		69%	335		78%	1029
	TP53:c.743G>A(p.(Arg248Gln))		NA	108		34%	282
	TP53:c.949dupC(p.(Gln317fs))		NA	106		35%	409
SEO11	CTNNB1:c.110C>T(p.(Ser37Phe))	70%	27%	22	60%	46%	78
SEO12	ARID1A:c.del544_557(p.(Ala182fs))	40%	37%	111	50%	39%	272
	ARID1A:c.3453delins(p.(Ser1151fs))		8%	341		37%	576
	PTEN:c.795_801+5delins(p.(?))		25%	112		56%	132
	TP53:c.818G>A(p.(Arg273His))		NA	946		61%	764
SEO13	ARID1A:c.2970G>T(p.(Lys990Asn))[a]	60%	NA	4	70%	33%	36
	ARID1A:c.3310G>T(p.(Glu1104*))		21%	95		NA	338
	ARID1A:c.3407C>T(p.(Ala1136Val))[a]		17%	153		NA	692
	ARID1A:c.4005-1G>T(p.(?))		NA	70		44%	234
	ARID1A:c.5225G>T(p.(Arg1742Ile))[a]		NA	36		33%	130
	CTNNB1:c.101G>A(p.(Gly34Glu))		39%	72		36%	222
	PIK3CA:c.263G>A(p.(Arg88Gln))		NA	18		46%	78
	POLE:c.857C>G(p.(Pro286Arg))		42%	86		36%	211
	PTEN:c.19G>T(p.(Glu7*))		43%	107		NA	436
	PTEN:c.239A>C(p.(Lys80Thr))[a]		NA	82		43%	447

	PTEN:c.1021T>G(p.(Phe341Val))[a]		NA	38		41%	263
	TP53:c.637C>T(p.(Arg213*))		NA	112		35%	349
	TP53:c.638G>A(p.(Arg213Gln))		30%	123		NA	348
SEO14	CTNNB1:c.94G>C(p.(Asp32His))	60%	38%	163	50%	21%	76
	PIK3CA:c.1624G>A(p.(Glu542Lys))		42%	200		NA	66
	PTEN:c.357dupC(p.(Ala120fs))		36%	167		10%	40
SEO15	ARID1A:c.3448dupA(p.(Thr1150fs))	70%	25%	16	50%	26%	423
	KRAS:c.35G>A(p.(Gly12Asp))		25%	8		22%	173
	PIK3CA:c.1031T>G(p.(Val344Gly))		NA	18		23%	243
	PTEN:c.73_74del(p.(Leu25fs))		11%	73		51%	890
SEO16	ARID1A:.c1934C>G(p.(Ser745*))	60%	86%	219	60%	NA	28
	CTNNB1:c.101G>T(p.(Gly34Val))		45%	227		NA	76
	CTNNB1:c.110C>A(p.(Ser37Tyr))		NA	226		26%	77
	PIK3CA:c.323G>A(p.(Arg108His))		43%	340		3%	67
	PIK3CA:c.3140A>G(p.(His1047Arg))		49%	188		NA	58
SEO17	ARID1A:c.4336C>T(p.(Arg1446*))	40%	13%	354	50%	NA	322
	ARID1A:c.5343_5344delins(p.(Val178fs))		23%	78		NA	74
	PTEN:c.1012delT(p.(Ser338fs))		31%	361		7%	294
SEO18	PIK3CA:c.3140A>G(p.(His1047Arg))	60%	29%	69	80%	42%	24
	TP53:c.159G>A(p.(Trp53*))		80%	30		91%	22
SEO19	PTEN:c.697C>T(p.(Arg233*))	70%	59%	1607	60%	64%	311
	TP53:c.711G>A(p.(Met237Ile))		88%	883		64%	197
SEO20	ARID1A:c.328_338(p.(Arg110fs))	50%	46%	1235	40%	34%	624
	ARID1A:c.1314_1337del(p.(Gln439fs))		15%	1420		13%	277
	PTEN:c.493-1G>T(p.(?))		30%	667		34%	335
	PTEN:c.919G>T(p.(Glu307*))		30%	1627		30%	723
SEO21	ARID1A:c.2187_2205del(p.(Gly731fs))	70%	17%	965	60%	40%	1573
	PTEN:c.1003C>T(p.(Arg335*))		45%	258		71%	380
SEO22	ARID1A:c.4003C>T(p.(Arg1335*))	60%	27%	63	70%	59%	44
	CTNNB1:c.94G>T(p.(Asp32Tyr))		22%	664		44%	236

	MTOR:c.5395G>A(p.(Glu1799Lys))		34%	235		37%	168
SEO23	TP53:c.289_307del(p.(Val97fs))	60%	60%	160	60%	78%	404
SEO24	ARID1A:c.3151C>G(p.(Leu1051Val))[a]	60%	15%	80	70%	54%	193
	CTNNB1:c.110C>T(p.(Ser37Phe))		20%	60		34%	203
	PTEN:c.49C>T(p.(Gln17*))		28%	221		88%	318
SEO25	TP53:c.241dupA(p.(Thr81fs))	30%	75%	40	60%	67%	24
SEO26	ARID1A:c.282delC(p.(Gly95fs))	60%	57%	60	80%	20%	20
	KRAS:c.38G>A(p.(Gly13Asp))		32%	559		56%	346
	PTEN:c.164+1_164+5del(p.(?))		42%	705		56%	486
	PTEN:c.388C>G(p.(Arg130Gly))		45%	1031		34%	724
SEO27	ARID1A:c.5164C>T(p.(Arg1722*))	40%	27%	807	60%	33%	1333
	PIK3CA:c.3140A>G(p.(His1047Arg))		23%	235		45%	609
	PTEN:c.512A>T(p.(Gln171Leu))[a]		30%	173		28%	300
	PTEN:c.1094_1100del(p.(Val365fs))		21%	181		34%	406
	TP53:c.529_546del(p.(delSer90_Thr91delinsThrArg))		21%	181		33%	133
	TP53:c.535C>T(p.(His179Tyr))		21%	229		39%	159
SEO28	PTEN:c.885_886del(p.(Cys296*))	30%	12%	742	30%	NA	364
	TP53:c.747G>T(p.(Arg249Ser))		NA	590		6%	276
	TP53:c.817C>T(p.(Arg273Cys))		5%	2577		NA	1189
SEO29	CTNNB1:c.109T>G(p.(Ser37Ala))	50%	15%	53	40%	NA	149
	PTEN:c.353dupA(p.(His118fs))		9%	92		NA	282
	PTEN:c.389G>A(p.(Arg130Gln))		59%	92		NA	282
	TP53:c.326delT(p.(Phe109fs))		NA	106		62%	203
SEO30	ARID1A:c.5483C>G(p.(Ser1828*))	30%	NA	8	50%	42%	210
	CTNNB1:c.110C>A(p.(Ser37Tyr))		50%	8		16%	263
	PIK3CA:c.1624G>A(p.(Glu542Lys))		15%	27		17%	412
	PTEN:c.388C>G(p.(Arg130Gly))		20%	10		23%	417
SEO31	ARID1A:c.1364dupG(p.(Gln456fs))	60%	2%	696	60%	NA	3667
	ARID1A:c.3397_3398insG(p.(Pro1132fs))		26%	2162		NA	4138
	ARID1A:c.3585del(p.(Asp1196fs))		NA	314		60%	1077

	KRAS:c.34G>T(p.(Gly12Cys))		21%	676		33%	2345
	PIK3CA:c.263G>A(p.(Arg88Gln))		NA	432		34%	1124
	PTEN:c.276C>A(.p.(Asp92Glu))		22%	673		37%	1930
	PTEN:c.388C>G(p.(Arg130Gly))		29%	1007		41%	3834
SEO32	ARID1A:c.1904G>T(p.(Arg635Ile))[a]	60%	5%	18	60%	NA	156
	ARID1A:c.2999C>A(p.(Ser1000Tyr))[a]		19%	105		NA	42
	ARID1A:c.3310G>T(p.(Glu1104*))		23%	261		NA	110
	PIK3CA:c.3129G>T(p.(Met1043Ile))		26%	131		24%	75
	POLE:c.857C>G(p.(Pro286Arg))		22%	218		31%	103
	PTEN:c.17A>C(p.(Lys6Thr))[a]		32%	250		22%	155
	PTEN:c.895G>T(p.(Glu299*))		16%	225		38%	84
	PTEN:c.1026+2T>G(p.(?))		22%	109		33%	82
SEO33	ARID1A:c.4268delC(p.(Pro1423fs))		22%	418		39%	144
	KRAS:c.35G>T(p.(Gly12Val))		19%	574		30%	157
	PIK3CA:c.1031T>G(p.(Val344Gly))		1%	813		20%	250
	PTEN:c.388C>G(p.(Arg130Gly))		30%	1273		57%	532
SEO34	ARID1A:c.4153G>A(p.(Glu1385Lys))[a]	60%	44%	391	70%	NA	599
	POLE:c.857C>G(p.(Pro286Arg))		38%	437		38%	984
	PTEN:c.19G>T(p.(Glu70*))		NA	953		43%	1521
	PTEN:c.169T>G(p.(Leu57Val))[a]		40%	865		40%	2305
	PTEN:c.424C>T(p.(Arg142Trp))[a]		44%	642		40%	1192
	PTEN:c.457G>T(p.(Asp153Tyr))		54%	1211		53%	2460
	PTEN:c.771C>A(p.(Phe257Leu))[a]		35%	343		31%	729
	PTEN:c.1038C>A(p.(Tyr346*))		45%	876		NA	1440
	PTEN:c.1091C>A(p.(Ser364Tyr))[a]		NA	308		43%	735
SEO35	CTNNB1:c.94G>A(p.(Asp32Asn))	70%	6%	690	70%	37%	141
	PTEN:c.209T>G(p.(Leu70Arg))		11%	709		91%	147
SEO36	CTNNB1:c.110C>T(p.(Ser37Phe))	70%	45%	996	70%	49%	131
SEO37	ARID1A:c.5498_5520del(p.(Arg1833fs))	60%	50%	80	50%	71%	132
	CTNNB1:c.122C>T(p.(Thr41Ile))		41%	273		40%	370

	PTEN:c.901_917(p.(Asp301fs))		86%	583		88%	798
SEO38	PIK3CA:c.1633G>A(p.(Glu545Lys))	30%	8%	1114	20%	2%	500
	PIK3CA:c.1634A>C(p.(Glu545Ala))		2%	1111		6%	140
SEO39	ARID1A:c.2077C>T(p.(Arg693*))	60%	68%	553	40%	77%	518
	CTNNB1:c.94G>T(p.(Asp32Tyr))		31%	884		43%	888
	PIK3CA:c.277C>T(p.(Arg93Trp))		36%	489		48%	446
	PTEN:c.254-1G>T(p.(?))		NA	715		43%	842
	PTEN:c.752G>T(p.(Gly251Val))[a]		59%	210		43%	228
SEO40	ARID1A:c.3977delC(p.(Prof1326fs))	70%	38%	211	50%	NA	204
	ARID1A:c.5548delG(p.(Asp1850fs))		NA	370		38%	184
	ARID1A:c.6420delC(p.(Phe2141fs))		NA	1694		39%	1021
	PIK3CA:c.263G>A(p.(Arg88Gln))		33%	1024		24%	833
	PIK3CA:c.1633G>A(p.(Glu545Lys))		NA	3145		34%	1988
	PTEN:c.301dupA(p.(Ile101fs))		24%	2792		33%	988
	TP53:c.1024C>T(p.(Arg342*))		21%	4724		NA	2742
SEO41	PTEN:c.945T>A(p.(Tyr315*))	60%	27%	200	70%	24%	300
	PTEN:c.1012delT(p.(Ser338fs))		38%	368		68%	750
	TP53:c.818G>A(p.(Arg273His))		37%	1449		63%	2800
SEO42	ARID1A:c.4555C>T(p.(Gln1519*))	70%	31%	858	50%	44%	536
	KRAS:c.35G>T(p.(Gly12Val))		47%	736		54%	663
	PTEN:c.295G>T(p.(Glu99*))		24%	513		25%	504
	PTEN:c.502_503del(p.(Ile168fs))		43%	302		56%	350
SEO43	ARID1A:c.526C>T(p.(Gln176*))	60%	30%	373	60%	37%	619
	ARID1A:c.3301dupT(p.(Tyr110fs))		20%	600		27%	1630
	KRAS:c.35G>C(p.(Gly12Ala))		26%	285		29%	1628
	PIK3CA:c.1633G>A(p.(Glu545Lys))		25%	496		30%	2333
	PTEN:c.738_739(p.(Leu247fs))		27%	96		28%	443
	PTEN:c.922_923ins(p.(Arg308fs))		28%	436		32%	1594
SEO44	ARID1A:c.5429A>G(p.(Asp1810Gly))[a]	50%	4%	88	90%	NA	844
	ARID1A:c.5965C>T(p.(Arg1989*))		39%	328		40%	605

	MTOR:c.5395G>A(p.(Glu1799Lys))		41%	546		44%	950
	PIK3CA:c.263G>A(p.(Arg88Gln))		43%	200		41%	707
	PIK3CA:c.3062A>G(p.(Tyr1021Cys))		47%	145		41%	571
	POLE:c.1231G>C(p.(Val411Leu))		45%	1347		44%	2225
	PTEN:c.38A>C(p.(Lys12Thr))[a]		40%	835		44%	1802
	PTEN:c.517C>T(p.(Arg173Cys))		54%	296		48%	925
	TP53:c.817C>T(p.(Arg273Cys))		44%	3025		42%	6310
	TP53:c.1182A>G(p.(*394Trpext*9))[a]		44%	823		45%	1240
SEO45	ARID1A:c.1554_1565del(p.(Tyr518_Pro522delins))	60%	18%	606	50%	33%	1009
	KRAS:c.35G>A(p.(Gly12Asp))		11%	527		37%	946
	PIK3CA:c.333G>C(p.(Lys111Asn))		17%	729		35%	1034
	PTEN:c.388C>G(p.(Arg130Gly))		51%	1445		87%	2672
SEO46	TP53:c.743G>A(p.(Arg248Gln))	60%	94%	1223	50%	59%	139
SEO47	KRAS:c.35G>T(p.(Gly12Val))	60%	40%	700	50%	42%	757
	PIK3CA:c.3129G>T(p.(Met1043Ile))		34%	659		26%	415
	PTEN:c.85delT(p.(Tyr29fs))		83%	489		69%	336
SEO48	PIK3CA:c.1634A>C(p.(Glu545Ala))	60%	1%	1000	60%	2%	471
	PIK3CA:c.1636C>A(p.(Gln546Lys))		4%	1027		NA	478
	PTEN:c.389G>T(p.(Arg130Leu))		7%	1465		NA	511
SEO49	ARID1A:c.4555delC(p.(Gln1519fs))	70%	27%	689	20%	NA	1135
	PIK3CA:c.1634A>C(p.(Glu545Ala))		NA	666		1%	2400
	PTEN:c.389delG(p.(Arg130fs))		63%	948		NA	1548
SEO50	KRAS:c.35G>A(p.(Gly12Asp))	30%	9%	391	40%	NA	274
	PIK3CA:c.1048G>A(p.(Asp350Asn))		8%	410		2%	353
MDEN1	ARID1A:c.1882dupA(p.(Met628fs))	30%	15%	187	50%	46%	691
	ARID1A:c.5386_5402del(p.(Pro1786*))		50%	60		63%	248
	KRAS:c.35G>T(p.(Gly12Val))		14%	29		89%	163
	MTOR:c.7280T>G(p.(Leu2427Arg))		16%	32		NA	308
	PTEN:c.1003delC(p.(Arg335fs))		15%	67		35%	223
	PTEN:c.1026+2T>A(p.(?))		22%	86		52%	219

MDEN2	PIK3CA:c.3140A>G(p.(His1047Arg))	70%	64%	175	50%	53%	392
	PTEN:c.289C>T(p.(Gln97*))		68%	76		50%	268
	PTEN:c.724G>T(p.(Glu242*))		21%	76		29%	97
MDEN3	PTEN:c.278A>C(p.(His78Pro))	65%	64%	119	40%	53%	64
	TP53:c.517G>A(p.(Val173Met))		77%	508		46%	191
MDEN4	TP53:c.916C>T(p.(Arg306*))	60%	79%	449	50%	48%	1677
MDEN5	ARID1A:c.2396delA(p.(Gln799fs))	40%	33%	673	20%	10%	313
	KRAS:c.38G>A(p.(Gly13Asp))		65%	206		15%	107
	PIK3CA:c.263G>A(p.(Arg88Gln))		33%	103		5%	42
	PTEN:c.469G>T(p.(Glu157Thr))		31%	626		11%	309
	PTEN:c.1027-5_1041del(p.(?))		35%	331		17%	141
MDEN6	TP53:c.151G>T(p.(Glu51*))	30%	83%	263	20%	89%	364
MDEN7	PIK3CA:c.3140A>G(p.(His1047Arg))	70%	64%	322	70%	85%	998
MDEN8	PIK3CA:c.1624G>A(p.(Glu542Lys))	60%	29%	6583	60%	28%	279
	TP53:c.80delC(p.(Pro27fs))		40%	223		59%	34
	TP53:c.733G>A(p.(Gly245Ser))		19%	4637		44%	164
MDEN9	ARID1A:c.2994delC(p.(Ser999fs))	70%	35%	1423	40%	21%	86
	ARID1A:c.3216delA(p.(Lys1072fs))		14%	1486		NA	148
	ARID1A:c.5548delG(p.(Asp1850fs))		8%	342		23%	26
	KRAS:c.35G>A(p.(Gly12Asp))		33%	2121		11%	127
	PIK3CA:c.263G>A(p.(Arg88Gln))		36%	917		19%	63
	PTEN:c.204C>A(p.(Tyr68*))		26%	2531		14%	157
	PTEN:c.388C>G(p.(Arg130Gly))		36%	3011		20%	280
MDEN10	MTOR:c.5395G>A(p.(Glu1799Lys))	60%	43%	1933	30%	36%	483
	PIK3CA:c.1637A>G(p.(Gln546Arg))		47%	3119		25%	900
	PTEN:c.278A>G(p.(His78Arg))		40%	1638		22%	373
	PTEN:c.517C>T(p.(Arg173Cys))		53%	1400		33%	364
MDEN11	KRAS:c.35G>A(p.(Gly12Asp))		42%	905		38%	284
	PTEN:c.209+1_209+2del(p.(?))		39%	1369		32%	525
	PTEN:c.330delA(p.(Glin110fs))		58%	1062		51%	357

MDEN12	TP53:c.320_340del(p.(Tyr107Phe113del))	80%	67%	321	40%	42%	107
MDEN13	ARID1A:c.2352_2353delins(p.(Met785fs))	40%	15%	3160	20%	5%	360
	ARID1A:c.4387C>G(p.(Arg1463Gly))[a]		4%	64		9%	18
	ARID1A:c.5954C>A(p.(Ser1985Tyr))[a]		18%	2511		9%	400
	PTEN:c.382A>G(p.(Lys128Glu))		26%	6954		18%	904
MDEN14	PIK3CA:c.316G>C(p.(Gly106Arg))	70%	35%	18997	60%	12%	2083
	TP53:c.670G>T(p.(Glu224*))		55%	3949		11%	736
MDEN15	TP53:c.586C>T(p.(Arg196*))	35%	10%	1000	20%	11%	1927
MDEN16	PIK3CA:c.1635G>T(p.(Glu545Asp))	50%	7%	4794	30%	2%	5059
	TP53:c.841G>C(p.(Asp281His))		7%	3855		2%	5059
MDEN17	PTEN:c.210-1G>A(p.(?))	40%	20%	7440	30%	7%	3515
	PTEN:c.693delC(p.(Thr232fs))		24%	2200		9%	848
	TP53:c.742C>T(p.(Arg248Trp))		24%	2458		9%	1000
MDEN18	PIK3CA:c.331A>G(p.(Lys111Glu))	70%	37%	2584	30%	12%	1817
	TP53:c.743G>A(p.(Arg248Gln))		56%	1629		11%	1473
MDEN19	ARID1A:c.3216delA(p.(Lys1072fs))	70%	41%	1620	70%	38%	705
	ARID1A:c.6067G>T(p.(Glu2023*))		35%	1997		32%	1050
	PIK3CA:c.263G>A(p.(Arg88Gln))		40%	885		31%	632
	PTEN:c.1delA(p.(?))		52%	3302		49%	1882
	PTEN:c.238A>G(p.(Lys80Glu))[a]		52%	5877		51%	3731
	PTEN:c.330_331del(p.(Trp111fs))		41%	3224		40%	2180
MDOV1	TP53:c.711G>T(p.(Met237Ile))	70%	74%	497	70%	89%	413
MDOV2	TP53:c.394A>C(p.(Lys132Gln))	40%	33%	248	40%	79%	84
MDOV3	TP53:c.536G>T(p.(Gly179Val))	60%	69%	374	60%	63%	400
MDOV4	TP53:c.400T>C(p.(Phe134Leu))[a]	60%	73%	485	70%	71%	663
MDOV5	TP53:c.671+1G>A(p.(?))	15%	29%	159	15%	22%	2545
MDOV6	TP53:c.673-3_683delins(p.(?))	60%	90%	2377	70%	85%	398
MDOV7	ARID1A:c.323A>G(p.(Thr1078Ala))[a]	50%	42%	267	80%	40%	555
	KRAS:c.35G>T(p.(Gly12Val))		52%	446		64%	695
	PTEN:c.202T>A(p.(Tyr68Asn))		22%	627		33%	1485

	PTEN:c.367C>T(p.(His123Tyr))		52%	615		63%	1457
MDOV8	TP53:c.517G>A(p.(Val173Met))	40%	57%	277	60%	71%	397
MDOV9	TP53:c.1024C>T(p.(Arg342*))	60%	92%	720	70%	93%	3504
MDOV10	TP53:c.493C>T(p.(Gln165*))	70%	92%	221	60%	83%	106
MDOV11	KRAS:c.35G>A(p.(Gly12Asp))	40%	11%	145	70%	64%	277
MDOV12	TP53:c.817C>T(p.(Arg273Cys))	60%	2%	476	70%	2%	353
MDOV13	ARID1A:c.2227C>T(p.(Gln743*))	80%	NA	2454	70%	26%	4642
	ARID1A:c.3222_3223del(p.(Glu1075fs))		NA	407		42%	1143
	ARID1A:c.3552dupA(p.(Val1185fs))		44%	186		NA	535
	PTEN:c.389G>A(p.(Arg130Gln))		62%	1044		51%	2249
	PTEN:c.416_440del(p.(Leu139fs))		36%	1750		25%	4092
MDOV14	TP53:c.645delT(p.(Ser215fs))	50%	70%	194	30%	66%	173
MDOV15	TP53:c.375+1G>A(p.(?))	40%	28%	150	40%	43%	502
MDOV16	TP53:c.1024delC(p.(Arg342fs))	50%	83%	193	50%	87%	1303
MDOV17	TP53:c.103_116del(p.(Leu35fs))	40%	9%	200	60%	49%	1249
MDOV18	TP53:c.733G>A(p.(Gly245Ser))	30%	22%	1636	40%	6%	2787
MDOV19	NA	10%	NA	NA	10%	NA	NA
MDOV20	PIK3CA:c.1258T>C(p.(Cys420Arg))	40%	8%	3114	30%	31%	1513
	TP53:c.817C>T(p.(Arg273Cys))		9%	5198		35%	2769

[a]Variants classified as class 3 (variant of unknown significance). All other variants were classified as class 4 or class 5.
MDEN, endometrial metastatic disease; MDOV, ovarian metastatic disease; NA, not applicable; SEO, synchronous endometrial and ovarian cancer; TCP, tumor cell percentage; VAF, variant allele frequent.

Supplementary Table 4. Frequency of cases harboring a shared vs. unique mutation, specified for each gene

	**Shared**	**Unique**	**Total**
ARID1A	65% (26/40)	35% (14/40)	40
CTNNB1	88% (14/16)	13% (2/16)	16
KRAS	93% (14/15)	7% (1/15)	15
MTOR	75% (3/4)	25% (1/4)	4
PIK3CA	88% (28/32)	12% (4/32)	32
POLE	100% (4/4)	0% (0/0)	4
PTEN	84% (42/50)	16% (8/50)	50
TP53	92% (35/38)	8% (3/38)	38

Supplementary Table 5.A Variants found per subgroup, including only carcinomas with endometrioid histology in both tumors

	SEO (n=32)	Endometrial MD (n=10)	Ovarian MD (n=4)	P[a]	P[b]
ARID1A					
No	18 (56.3)	6 (60.0)	4 (100)	1.000	0.141
Yes	14 (43.8)	4 (40.0)	0 (0)		
CTNNB1					
No	19 (59.4)	10 (100)	4 (100)	**0.018**	0.274
Yes	13 (40.6)	0	0		
KRAS					
No	28 (87.5)	7 (70.0)	4 (100)	0.328	1.000
Yes	4 (12.5)	3 (30.0)	0		
MTOR					
No	30 (93.8)	9 (90.0)	4 (100)	1.000	1.000
Yes	2 (6.3)	1 (10.0)	0		
PIK3CA					
No	20 (67.5)	4 (40.0)	4 (100)	0.281	0.278
Yes	12 (37.5)	6 (60.0)	0		
POLE					
No	30 (93.8)	10 (100)	4 (100)	1.000	1.000
Yes	2 (6.3)	0	0		
PTEN					
No	10 (31.1)	2 (20.0)	3 (75.0)	0.696	0.124
Yes	22 (68.8)	8 (80.0)	1 (25.0)		
TP53					
No	28 (87.5)	8 (80.0)	1 (25.0)	0.616	**0.018**
Yes	4 (12.5)	2 (20.0)	3 (75.0)		

P-values were obtained using the Fisher's exact test and χ^2. Values are presented as median (range) or number (%).
SEO, synchronous endometrial and ovarian cancer; MD, metastatic disease.
[a]P<0.05 comparing SEO with endometrial MD.
[b]P<0.05 comparing SEO with ovarian MD.

Supplementary Table 5.B Variants found per subgroup, including only carcinomas with non-endometrioid histology in one or both tumors

	**SEO (n=18)**	**Endometrial MD (n=9)**	**Ovarian MD (n=16)**	**P[a]**	**P[b]**
ARID1A					
No	12 (66.7)	8 (88.9)	15 (93.8)	0.363	0.090
Yes	6 (33.3)	1 (11.1)	1 (6.3)		
CTNNB1					
No	17 (94.4)	9 (100)	16 (100)	1.000	1.000
Yes	1 (5.6)	0	0		
KRAS					
No	14 (77.8)	8 (88.9)	14 (87.5)	0.636	0.660
Yes	4 (22.2)	1 (11.1)	2 (12.5)		
MTOR					
No	18 (100)	9 (100)	16 (100)	-	-
Yes	0	0	0		
PIK3CA					
No	13 (72.2)	5 (55.6)	15 (93.8)	0.423	0.180
Yes	5 (27.8)	4 (44.4)	1 (6.3)		
POLE					
No	16 (88.9)	9 (100)	16 (100)	0.538	0.487
Yes	2 (11.1)	0	0		
PTEN					
No	10 (55.6)	7 (77.8)	15 (93.8)	0.406	**0.019**
Yes	8 (44.4)	2 (22.2)	1 (6.3)		
TP53					
No	13 (72.2)	1 (11.1)	3 (18.8)	**0.004**	**0.003**
Yes	5 (27.8)	8 (88.9)	13 (81.3)		

P-values were obtained using the Fisher's exact test and χ^2. Values are presented as median (range) or number (%).
SEO, synchronous endometrial and ovarian cancer; MD, metastatic disease.
[a]P<0.05 comparing SEO with endometrial MD.
[b]P<0.05 comparing SEO with ovarian MD.

Supplementary Table 6. The Cancer Genome Atlas analysis to compare mutational profiles from the publicly available TCGA endometrial carcinoma tumor set with synchronous endometrial and ovarian cancers. Both groups were stratified by histological subtype (endometrioid left, non-endometrioid right).

	**Endometrioid**			**Non-endometrioid**		
	SEO (%) N = 32	TCGA (%) N = 193	P-value	SEO (%) N = 18	TCGA (%) N = 43	P-value
ARID1A	44	39	0.600	33	9	0.051
CTNNB1	40	41	0.974	6	0	0.295
KRAS	13	23	0.247	22	2	**0.024**
MTOR	6	13	0.386	0	2	1.000
PIK3CA	38	52	0.120	28	33	0.771
POLE	6	13	0.386	11	2	0.205
PTEN	69	78	0.238	44	2	**<0.001**
TP53	13	14	1.000	28	88	**<0.001**

P-values were obtained using the Fisher's exact test and χ^2.

SEO, synchronous endometrial and ovarian cancer; TCGA, The Cancer Genome Atlas.
